# Deficiency of MSH2 expression is associated with clear cell renal cell carcinoma

**DOI:** 10.3892/ol.2014.2482

**Published:** 2014-08-26

**Authors:** KOO HAN YOO, KYU YEOUN WON, SUNG-JIG LIM, YONG-KOO PARK, SUNG-GOO CHANG

**Affiliations:** 1 Department of Urology, School of Medicine, Kyung Hee University, Seoul 130-702, Republic of Korea; 2 Department of Pathology, School of Medicine, Kyung Hee University, Seoul 130-702, Republic of Korea

**Keywords:** renal cell carcinoma, DNA methylation, MSH2 protein, immunohistochemistry

## Abstract

DNA hypermethylation plays a major role in the regulation of gene expression in differentiation, development and diseases. The DNA mismatch repair system, which includes Mut-S-Homologon-2 (MSH2) protein, is essential to maintain the stability of the genome during repeated duplication. This study aimed to investigate tumoral MSH2 immunohistochemical expression in clear cell renal cell carcinoma (RCC), and the associations between tumoral MSH2 immunohistochemical expression and clinicopathological parameters. Previously, we reported a high-throughput method for analyzing the methylation status of 807 preselected genes; Illumina’s GoldenGate Methylation Cancer Panel I microarray. The MSH2 gene was identified to be hypermethylated in cancer tissue compared with normal tissue. From January 2000 to December 2012, 129 clear cell RCC cases (median age, 61 years) were included in the immunohistochemical analysis of the present study. Patients were divided according to MSH2 expression status (MSH2-negative, n=53; MSH2-positive, n=76). T stage was significantly higher in the MSH2-negative group than in the MSH2 positive-group (P=0.021). There was no significant difference in terms of N stage, M stage and Fuhrman’s nuclear grade between the MSH2-negative and MSH2-positive group (N stage, P=0.072; M stage, P=0.759; Fuhrman’s nuclear grade, P=0118). The MSH2-negative group showed decreased rates of recurrence-free survival, progression-free survival and overall survival, without statistically significant results (P=0.232, P=0.268 and P=0.311, respectively). MSH2 protein expression may be a useful marker for predicting TNM stage and prognosis and, thus, MSH2 may be a prognostic factor in clear cell RCC.

## Introduction

Renal cell carcinoma (RCC) is the most common neoplasm in the kidney, with an estimated 5-year survival rate of 50–60%. In the United States, RCC incidence has been increasing with an estimated 65,150 new cases and 13,680 deaths in 2013 (1). In the Republic of Korea, RCC accounts for ~1% of all primary malignancies and is the 10th most common cancer in males (2). RCC has several subtypes, each derived from various parts of the nephron, with each presenting different genetic characteristics, histological features and clinical phenotypes. The most common subtype is the clear cell type, accounting for >75% of cases (3). Recent research in genetics has enabled the discovery of alterations in different pathways and the benefits of molecular profiling research have already been incorporated into clinical oncology, such as targeted therapy (4). Development of targeted therapies has changed the treatment of metastatic RCC, and further information will become available from the genomic approach to tumor classification, prognostic markers and predictive indicators of response to treatment, along with personal susceptibility to developing cancer when exposed to risk factors (5).

DNA hypermethylation plays a critical role in the regulation of gene expression in differentiation, development and disease (6). Changes in DNA methylation are recognized as one of the most common forms of molecular alteration in cancer development. Hypermethylation of CpG islands located in the promoter regions of tumor suppressor genes is established as the most frequent mechanism for gene inactivation. Previously, we reported the hypermethylation status of the Mut-S-Homologon-2 (MSH2) gene in clear cell RCC (7). A high-throughput genotyping assay (GoldenGate Methylation Cancer Panel I microarray; Illumina, San Diego, CA, USA) (6) was adapted to determine the methylation status of 1,505 specific CpG sites in 807 cancer-related genes. Tissue specimens consisted of 62 cancer tissues and 62 matched adjacent normal tissues obtained from clear cell RCC patients of the Kyung Hee University Hospital (Seoul, Korea). The results revealed that the mean β-value difference between cancer and normal tissues was 0.30±0.28 for MSH2. We examined the methylation status of CpG sites by bisulfite sequencing. The results showed that the methylation rate of the MSH2 gene was 54.8% in cancer tissue and 26.1% in normal tissue. The MSH2 gene was hypermethylated in cancer tissue compared with normal tissue.

The DNA mismatch repair (MMR) system is essential to maintain the stability of the genome during repeated duplication (8). Main functions of the MMR system include the correction of biosynthetic errors, DNA damage surveillance and prevention of recombination. The MMR system is composed of a few well-conserved proteins, including Mut-S-Homolog proteins and Mut-L-Homolog proteins. Genetic studies have revealed that MSH2, which is one of the Mut-S-Homolog proteins, is required for all mismatch corrections in nuclear DNA during replication; whereas Mut-S-Homolog-3 and Mut-S-Homolog-6, which are also Mut-S-Homolog proteins, are required for the repair of certain overlapping types of mismatched DNA (9).

In present study aimed to investigate tumoral MSH2 immunohistochemical expression in clear cell RCC, as well as the associations between tumoral MSH2 immunohistochemical expression and clinicopathological parameters.

## Material and methods

### Patients and tissue specimens

Tissue samples from 129 clear cell RCC cases, 88 males (median age, 61.0±12.31 years) and 41 females (median age, 60.0±10.97 years), were used. All neoplasms were surgically resected at the Kyung Hee University Hospital and the Kyung Hee University Hospital at Gangdong (Seoul, Korea) from January 2000 to December 2012. Tumors were graded according to criteria of the American Joint Committee on Cancer (10). The clinical parameters, including tumor grade, recurrence, progression and overall survival, were analyzed along with the immunohistochemical results. The institutional review board of the Kyung Hee University Hospital at Gangdong approved this study (KHNMC IRB 2013-040). Written informed consent was obtained from all patients.

### Immunohistochemical staining

**Figure 1:**
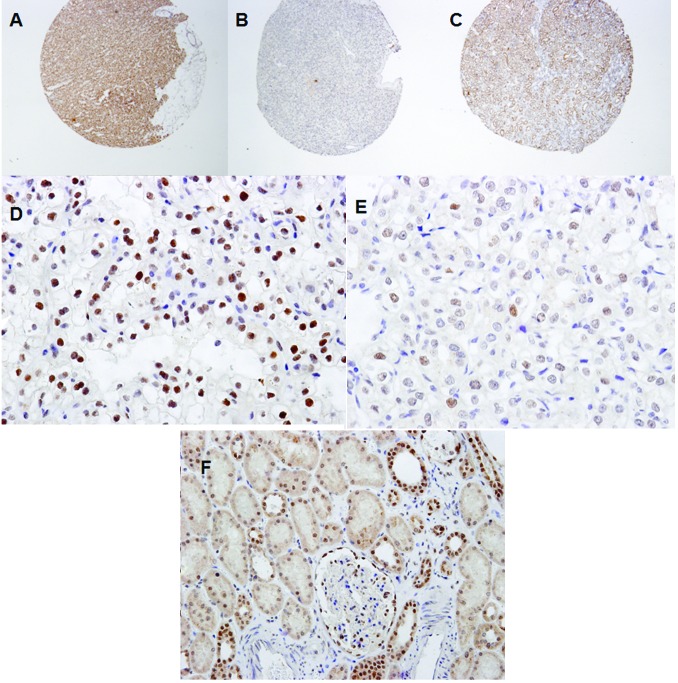
Tissue microarrays using clear cell RCC and normal kidney tissue. (A) Diffusely strong positive expression of MSH2 in clear cell RCC tissues at low magnification (x20). (B) Negative expression of MSH2 in clear cell RCC tissues at low magnification (x20). (C) Normal kidney cells show diffusely strong MSH2 expression at low magnification (x20). (D) RCC cells show diffusely strong nuclear MSH2 expression (magnification, ×200). (E) RCC cells show focally weak nuclear MSH2 expression (magnification, ×200). (F) MSH2 protein is expressed in the nuclei of tubular epithelial cells and parietal cells of Bowman’s capsule (magnification, ×100). RCC, renal cell carcinoma.

The tissue microarrays were assembled using a commercially available manual tissue microarrayer (Quick-Ray; Unitma Co., Ltd., Seoul, Korea) (11). Three representative tumor cores with diameters of 2.0 mm were punched from each tumor tissue block. Each of the tissue microarray blocks contained three normal kidney tissue cores. Immunohistochemistry was performed on 4-μm tissue sections from each tissue microarray block using the Bond Polymer Intense Detection system (Vision BioSystems, Mount Waverley, Victoria, Australia). Sections were incubated for 15 min at ambient temperature with primary mouse anti-human MSH2 monoclonal antibody (1:3,000, clone G219-1129; BD Biosciences, San Jose, CA, USA), using a biotin-free polymeric horseradish peroxidase-linker antibody conjugate system in a Bond-max automatic slide stainer (Vision BioSystems). Nuclei were counterstained with hematoxylin. The negative control was treated in an identical manner using mouse IgG instead of the primary antibody. The degree of expression by immunohistochemistry was classified by three pathologists blinded to the data. Semiquantitative analysis of immunoreactivity was performed according to intensity and proportion. The intensity score was determined as 0, no staining; 1, weak but detectable staining; 2, distinct staining; and 3, strong staining. The proportion score was determined as 1, 0–10%; 2, 11–50%; 3, 51–80%; and 4, 81–100%. The total score was the sum of the intensity score and the proportion score. Total scores were as follows: 1 and 2, negative staining; and 3–7, positive staining (Fig. 1A–C) (12–14).

### Statistical analysis

The χ*^2^* test and linear-by-linear association were used to evaluate the association between the degree of expression determined by immunohistochemistry (negative staining group, MSH2-negative group; positive staining group, MSH2-positive group) with clinicopathological variables. Survival was estimated using the Kaplan-Meier method, and comparisons among survival curves were made using the log-rank test. P<0.05 was considered to indicate a statistically significant difference. Statistical analyses were performed using SPSS software, version 16 (SPSS, Inc., Chicago, IL, USA).

## Results

### Immunohistochemical expression pattern of clear cell renal cell carcinoma

In the cancer tissue, nuclear MSH2 protein expression was diffusely strong (Fig. 1D) or focally weak (Fig. 1E). Negative and positive MSH2 expression was identified in 53 (41.1%) and 76 (58.9%) out of 129 cases, respectively. In the normal tissue, MSH2 protein was expressed in the nuclei of tubular epithelial cells and parietal cells of Bowman’s capsule (Fig. 1F).

### Associations between MSH2 protein expression and clinical variables

Overall, 129 clear cell RCC cases (median age, 61 years) were included in the study. The comparison between MSH2-negative patients (n=53) and MSH2-positive patients (n=76) is shown in Table I. There were 35 males and 18 females in MSH2-negative group, and 53 males and 23 females in MSH2-positive group (P=0.657). The median patient age (±SD) was 61.00±11.06 years in the MSH2-negative group and 60.00±12.50 years in the MSH2-positive group (P=0.823).

T stage was significantly higher in the MSH2-negative group than in the MSH2-positive group (P=0.021). There were no significant differences in terms of N stage, M stage and Fuhrman’s grade between the two groups (N stage; P=0.072, M stage; P=0759, Fuhrman grade; P=0118).

### Kaplan-Meier survival analysis

**Figure 2:**
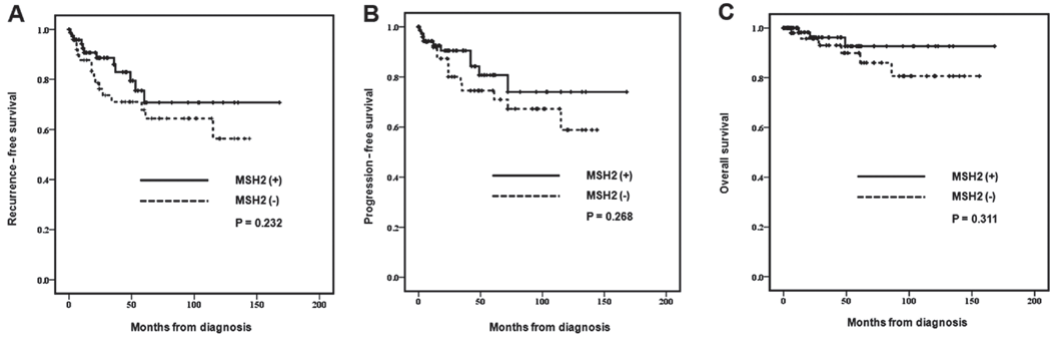
(A) Recurrence-free survival, (B) progression-free survival and (C) overall survival of the 129 clear cell renal cell carcinoma patients were analyzed according to the degree of expression by immunohistochemistry.

The MSH2-negative group showed decreased rates of recurrence-free survival, progression-free survival and overall survival, without statistically significant results (P=0.232, P=0.268 and P=0.311, respectively) (Fig. 2).

## Discussion

DNA methylation in cancer has been recognized for over 30 years (15–16). Two mechanisms of losses of methylation for carcinogenesis have been proposed. A weakening of transcriptional repression in normally silent regions could cause the harmful expression of inserted viral genes and repeat elements, and of normally silenced genes (17–18). Losses of methylation may affect the functional stability of chromosomes in cancer (18). Pericentromeric regions of chromosomes depend on correct levels of DNA methylation for the stability of DNA. As hypermethylation in gene promoters occurs early on in tumor progression, inhibiting or reversing these changes has a potential benefit for cancer prevention (20). Reactivation of the types of genes that are epigenetically silenced in cancer may have a profound antitumor effect. Early detection of cancer is essential to survival of cancer patients. The use of hypermethylation of CpG islands as tumor markers has benefited from extremely sensitive polymerase chain reaction (PCR) methods to detect methylated DNA sequences (16).

Several studies have reported associations between MSH2 gene methylation and gastrointestinal and urothelial tumor carcinogenesis (15,21,22). Heritable germline epimutations in MSH2 have been identified in a small number of Lynch syndrome families, which did not exhibit germline mutations in the MSH2 gene (23). Nagasaka *et al* reported the methylation status of the MSH2 gene in 268 colorectal cancers tissues, which comprised 222 sporadic colorectal cancers and 46 Lynch syndrome tumors that did not express MSH2 (24). The results showed that there was frequent MSH2 hypermethylation in Lynch syndrome tumors with MSH2 deficiency. The authors concluded that high levels of aberrant methylation at CpG island methylator phenotype-related markers in MSH2-methylated tumors highlights the possibility that MSH2 is a target which is susceptible to aberrant methylation in Lynch syndrome. Tumor-specific alterations of MSH2 hypermethylation in circulating DNA have been associated with esophageal squamous cell carcinoma (25). Promoter hypermethylation of MSH2 was analyzed using real-time methylation-specific PCR in paired tumor and plasma samples of 209 patients with esophageal squamous cell carcinoma. Aberrant MSH2 methylation was found in 101 of 209 esophageal squamous cell carcinoma patients. Follow-up analysis indicated significantly lower disease-free survival rates for patients with high MSH2 methylation compared with those with MSH2 unmethylation after surgery.

Recently, it was reported that patients with Lynch syndrome and MSH2 mutation are at an increased risk of developing urothelial carcinoma (26). Using cancer data of the Familial Gastrointestinal Cancer Registry in Toronto, Lynch syndrome patients with MSH2 mutations are at an increased risk of developing not only upper tract urothelial cancer but also bladder cancer, and could be offered appropriate screening (26). Stoehr *et al* reported that MMR proteins hMLH1 and hMSH2 are differentially expressed in the three main subtypes of sporadic RCC (clear cell, papillary and chromophobe) (27). Expression of hMLH1 and hMSH2 was investigated in 166 RCC patients of the main subtypes (101, clear cell; 30, papillary; 32, chromophobe; and 3, mixed RCC) by immunohistochemistry. Expression of hMLH1 and hMSH2 was reduced in 83.7% (118/141) and 51.2% (65/127) of clear cell RCC and papillary RCC cases, respectively. None of the clear cell RCC tumors showed high hMLH1 expression, while papillary and chromophobe RCC demonstrated preserved high hMLH1 expression in 25.0 and 33.3% of cases, respectively. Subtype specificity was present in the hMSH2 staining; chromophobe RCC showed high expression in 41.7% of cases, while clear cell and papillary tumors did not retain high expression. Therefore, diminished MMR protein expression was linked to tumor entity and may contribute to the different biological behavior of the RCC subtypes.

Previously, we utilized a high-throughput method for analyzing the methylation status of 807 preselected genes; the GoldenGate Methylation Cancer Panel I microarray (6,7). The results revealed that the mean β-value difference between cancer and normal tissues was 0.30±0.28 for MSH2, and the methylation rate of the MSH2 gene was 54.8% in cancer tissue and 26.1% in normal tissue. The MSH2 gene was hypermethylated in cancer tissue compared with normal tissue. In the present study, using immunohistochemical staining, T stage was found to be significantly higher in MSH2-negative clear cell RCC patients, compared with those that were MSH2-positive. There was no significant difference in terms of N stage, M stage and Fuhrman’s grade between the MSH2-negative and MSH2-positive group. The MSH2-negative group showed decreased rates of recurrence-free survival, progression-free survival and overall survival, without statistically significant results. The product of the MSH2 gene corrects any mismatched nucleotide errors in the DNA strand using the mismatch-repair mechanism and maintains the precise fidelity of DNA replication. Chen *et al* (28) reported a new primary RCC cell line and revealed a deletion of 1476 bp encoding 492 amino acids of MSH2 cDNA, truncated forms of MSH2, and microsatellite instability using a clear cell RCC cell line. Chen *et al* concluded that genomic instability caused by a variety of mutational events appears to be the important cornerstone of carcinogenesis. However, Leach *et al* reported that complete inactivation of mismatch repair is uncommon in RCC (29). Leach *et al* demonstrated that genetic alterations affecting expression were limited to MLH1 since MSH2, MSH6, PMS2 were detectable in their RCC cell lines. Chen *et al* and Leach *et al* reported that a damaged mismatch repair system is related to RCC (28,29). In order to demonstrate this outcome, one of the genetic methods utilized were microsatellite mutations. In our study, we observed similar results using DNA methylation. We believe that this indicates that the results are not only due to genetic factors, such as mutations, but also epigenetic factors, such as DNA methylation.

In conclusion, the current study identified that MSH2-negative staining was associated with a higher T stage in clear cell RCC patients. MSH2 protein expression may be a useful marker for predicting TNM stage and prognosis and, thus, MSH2 may be a prognostic factor in clear cell RCC. The results of the present study may lead to further large-scale studies regarding the identification of tumor markers for clear cell RCC.

## Figures and Tables

**Table I:** Analysis of MSH2 immunohistochemistry results and clinicopathological parameters of 129 clear cell renal cell carcinoma patients.

Parameter	MSH2-negative group (n=53, %)	MSH2-positive group (n=76, %)	P-value
Males/females, n	35/18	53/23	0.703^a^
Median age, years (±SD)	61.00±11.06	60.00±12.50	0.823^b^
Clinical T stage, n			0.021^c^
T1a	17 (32)	43 (57)	
T1b	14 (26)	16 (21)	
T2a	9 (17)	6 (8)	
T2b	1 (2)	2 (3)	
T3a	9 (17)	7 (9)	
T3b	1 (2)	2 (3)	
T3c	0 (0)	0 (0)	
T4	1 (2)	0 (0)	
Clinical N stage, n			0.072^a^
N0	49 (92)	75 (99)	
N1	4 (8)	1 (1)	
Clinical M stage, n			0.759^a^
M0	48 (91)	70 (92)	
M1	5 (9)	6 (8)	
Fuhrman’s nuclear grade, n			0.118^c^
Grade I	3 (6)	4 (5)	
Grade II	20 (38)	40 (53)	
Grade III	22 (42)	26 (34)	
Grade IV	8 (15)	6 (8)	

a Fisher’s exact test,

b Independent T-test,

c Linear by linear association test.
